# Massive Upper Gastrointestinal Hemorrhage in Brunner’s Gland Hamartoma of Duodenum

**DOI:** 10.7759/cureus.15875

**Published:** 2021-06-23

**Authors:** Arkadeep Dhali, Sukanta Ray, Ranajoy Ghosh, Avik Sarkar, Gopal Krishna Dhali

**Affiliations:** 1 Department of Gastrointestinal Surgery, School of Digestive and Liver Diseases, Institute of Postgraduate Medical Education and Research, Kolkata, IND; 2 Department of Gastrointestinal Pathology, School of Digestive and Liver Diseases, Institute of Postgraduate Medical Education and Research, Kolkata, IND; 3 Department of Gastrointestinal Radiology, School of Digestive and Liver Diseases, Institute of Postgraduate Medical Education and Research, Kolkata, IND; 4 Department of Gastroenterology, School of Digestive and Liver Diseases, Institute of Postgraduate Medical Education and Research, Kolkata, IND

**Keywords:** brunner’s gland hamartoma, massive upper gastrointestinal hemorrhage, gastric outlet obstruction, polypectomy, endoscopy

## Abstract

Brunner's gland hamartoma (BGH) is a rare benign small bowel tumor, mostly encountered in the duodenum. Massive upper gastrointestinal (UGI) hemorrhage is an unusual presentation rarely reported in English literature. Symptomatic patients mostly present with features of gastric outlet obstruction, occult bleeding, or intussusception. Herein, we report a case of BGH presenting with overt UGI bleed and features of gastric outlet obstruction. Esophagogastroduodenoscopy revealed a smooth polypoidal swelling in the posterior wall of the duodenal bulb. An endoscopic ultrasound (EUS) guided fine-needle-aspiration was performed, which was inconclusive. Contrast-enhanced computed tomography showed the absence of any extraluminal component of the lesion. Endoscopic polypectomy was attempted but failed due to the broad base of the lesion, and hence the patient was managed by open surgical excision. Histological examination of the resected specimen confirmed the diagnosis to be BGH. The patient had an uneventful recovery and was doing well at the 15-month follow-up. BGH should be considered as a differential diagnosis of a polypoidal lesion of the duodenum. Any lesion larger than 2 cm or symptomatic should be removed either by endoscopic or surgical intervention.

## Introduction

Brunner’s gland hamartoma is a rare tumor with an incidence of <0.01% accounting for approximately 5-10% of benign duodenal tumors [1]. Usually asymptomatic, it may present with upper gastrointestinal hemorrhage or gastric outlet obstruction. Endoscopy has both diagnostic as well as therapeutic implications. Although surgical resection is reserved for cases where endoscopic snare dissection is either not possible or has failed. This was seen in our case due to the broad base of the tumor.

## Case presentation

**Figure 1:**
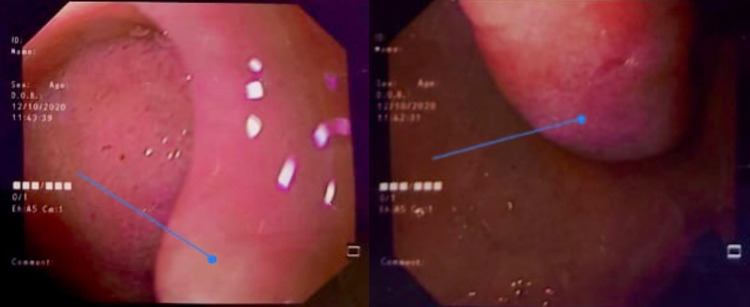
Esophagogastroduodenoscopy image showing a smooth sessile polypoidal swelling with a large base in the first part of the duodenum measuring >2 cm in the posterior wall of the duodenal bulb with no ulcer or bleeding

**Figure 2:**
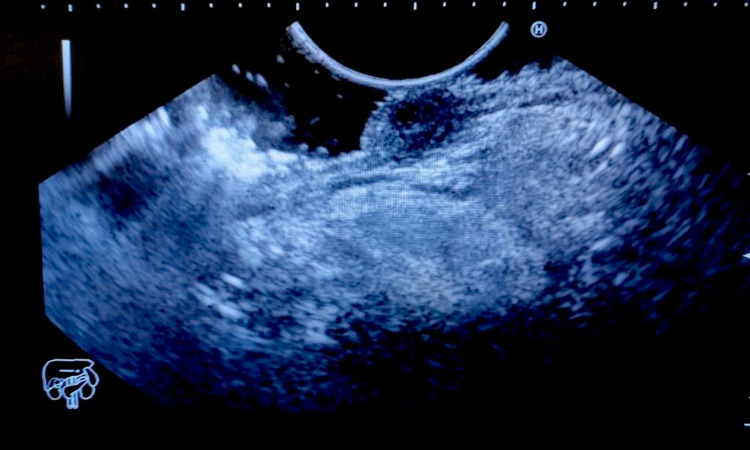
EUS showing a 2.2 cm hyperechoic lesion arising from the second layer (submucosa) with no calcification, cystic change, or ductal structure EUS (Endoscopic Ultrasound)

**Figure 3:**
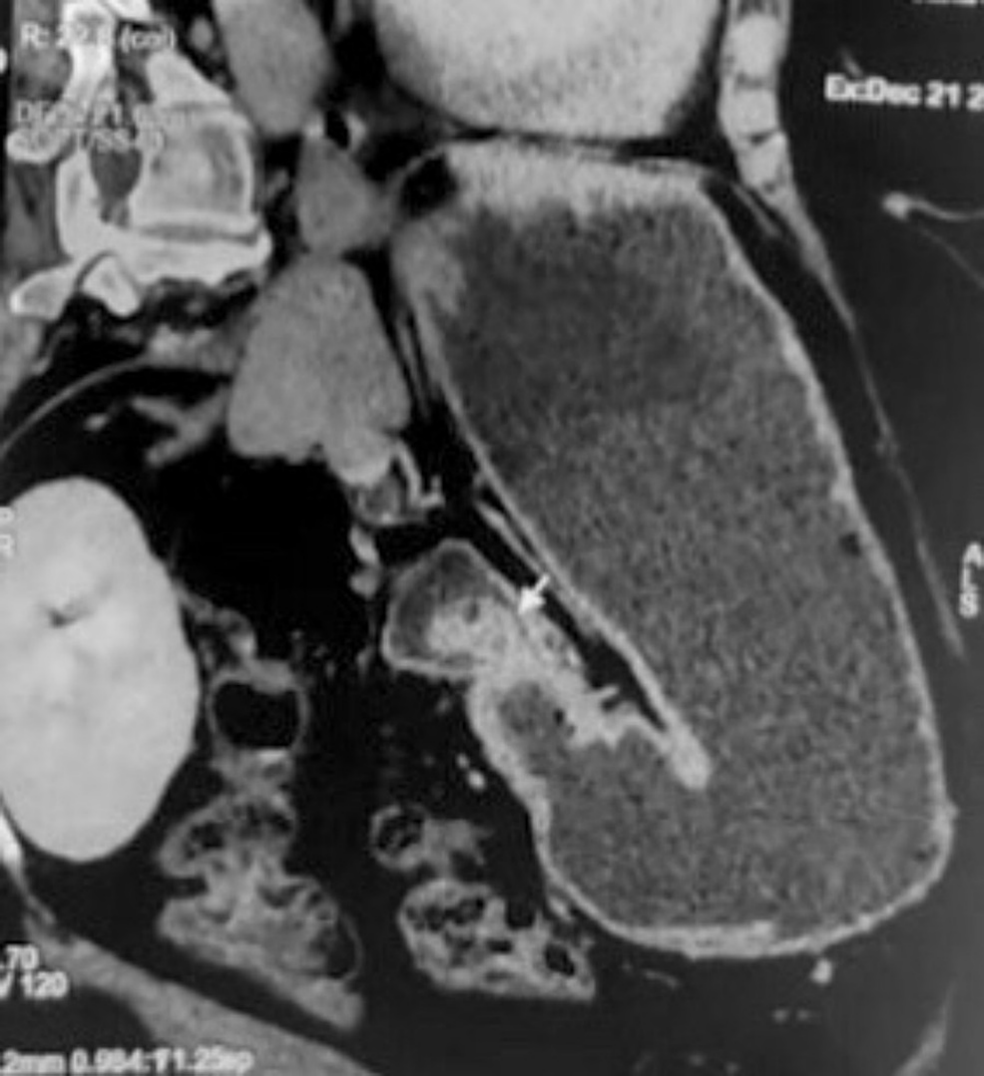
Contrast-enhanced computed tomography showing a homogeneously enhancing polypoidal mass arising from the posterior wall of duodenal bulb measuring 2.3 x 1.5 x 1.5 cm with no periduodenal fat stranding

**Figure 4:**
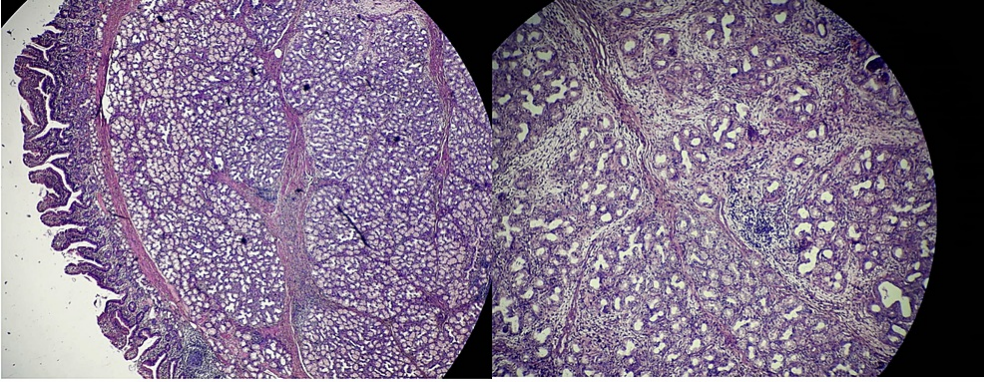
H&E image showing an admixture of fibrovascular tissue, adipose tissue, and hyperplastic Brunner’s gland extending to lamina propria from submucosa suggestive of BGH H&E: Hematoxylin and eosin BGH: Brunner's gland hamartoma

 A 44-year-old female presented with a history of hematemesis and melena for seven days. She also complained of multiple episodes of non bilious vomiting for the last eight months associated with mild abdominal discomfort and loss of appetite. Pallor was present on physical examination. Her hemoglobin was 7 gm/dL and was treated with two units of blood transfusion. Post-transfusion hemoglobin was 11 gm/dL. Esophagogastroduodenoscopy revealed a smooth sessile polypoidal swelling with a large base in the first part of the duodenum measuring >2 cm in the posterior wall of the duodenal bulb with no ulcer or bleeding (Figure 1). To characterize the lesion better, endoscopic ultrasound (EUS) was performed (Figure 2), which revealed a 2.2 cm hyperechoic lesion arising from the second layer (submucosa) with no calcification, cystic change, or ductal structure. A EUS guided fine-needle-aspiration was done, which was inconclusive. To look for any exophytic component of the lesion, contrast-enhanced computed tomography was performed, which showed a homogeneously enhancing polypoidal mass arising from the posterior wall of the duodenal bulb measuring 2.3 x 1.5 x 1.5 cm with no periduodenal fat stranding (Figure 3). Endoscopic polypectomy was attempted but failed due to the broad base of the lesion and compromised luminal size, and hence surgical intervention was warranted. After taking informed consent, she was taken up for open polypectomy with pyloroplasty. Intra-operatively after partial kocherization, the duodenal cap was opened longitudinally, and a sessile polyp measuring approximately 2.5 x 2 cm was removed after transfixing its base. The mucosal gap was repaired. The longitudinal incision was extended towards the pyloric antrum and closed transversely. The resected specimen was sent for histopathological examination, which showed an admixture of fibrovascular tissue, adipose tissue, and hyperplastic Brunner’s gland extending to lamina propria from submucosa suggestive of Brunner's gland hamartoma (BGH) (Figure 4). The postoperative period was uneventful, and she was doing well on a 15-month follow-up. 

## Discussion

Brunner’s glands are located in the submucosa of the proximal duodenum, which secretes alkaline mucin and protects duodenal mucosa from gastric chyme [2,3]. Brunner’s gland hamartoma (BGH), previously classified as glandular adenoma, is an admixture of tissues including smooth muscle, adipose tissue, lymphoid tissue, duct, acini, and smooth sheets of Brunner’s gland [3].

These are generally solitary, sessile, or pedunculated polypoidal lesions with equal sex distribution, diagnosed in the fifth decade of life, found in the posterior wall of the duodenal bulb [4,5]. Mostly they are asymptomatic but may present with features of upper gastrointestinal (UGI) hemorrhage or gastric outlet obstruction, both of which were present in our case. The UGI hemorrhage is mostly occult, with a majority of the patients presenting with anemia or chronic blood loss. In a study by Levine et al., 37% of the patients had UGI bleed, and 37% had obstructive symptoms [5]. Atypical clinical features include obstructive jaundice, chronic diarrhea, and pancreatitis [2]. Massive UGI hemorrhage is an unusual presentation rarely reported in English literature.

Diagnosis is made by radiological and endoscopic findings. Endoscopic biopsy findings are often non-contributory as they cannot reach deep submucosal tumor tissue [6,7]. Contrast-enhanced computed tomography rules out extraluminal extension of the lesion.

Treatment regarding asymptomatic, incidentally found BGH is controversial. Larger lesions (>2 cm) are shown to complicate with acute bleeding leading to shock [5,8]. Hence even if they are asymptomatic, they require treatment. All symptomatic patients require treatment. It can be either endoscopic snare-polypectomy or surgical excision. For a small, pedunculated lesion, endoscopic management is preferred, but in failed endoscopic management or in cases where endoscopy is not possible, surgical management is warranted.

## Conclusions

Brunner’s gland hamartoma is an important differential to be considered in polypoidal lesions of the duodenum. Removal of lesions, either by endoscopic or surgical methods, is required in all symptomatic cases and asymptomatic cases where the size of the lesion is >2 cm. The endoscopic snare cautery technique, although more cost-effective and less invasive, is often not possible if the polyp is large enough or has a large base. In these cases, surgical excision is the treatment of choice.
